# SHMT1 knockdown induces apoptosis in lung cancer cells by causing uracil misincorporation

**DOI:** 10.1038/cddis.2014.482

**Published:** 2014-11-20

**Authors:** A Paone, M Marani, A Fiascarelli, S Rinaldo, G Giardina, R Contestabile, A Paiardini, F Cutruzzolà

**Affiliations:** 1 Department of Biochemical Sciences, ‘Sapienza' University of Rome, Rome, Italy

## Abstract

Reprogramming of cellular metabolism towards *de novo* serine production fuels the growth of cancer cells, providing essential precursors such as amino acids and nucleotides and controlling the antioxidant and methylation capacities of the cell. The enzyme serine hydroxymethyltransferase (SHMT) has a key role in this metabolic shift, and directs serine carbons to one-carbon units metabolism and thymidilate synthesis. While the mitochondrial isoform of SHMT (SHMT2) has recently been identified as an important player in the control of cell proliferation in several cancer types and as a hot target for anticancer therapies, the role of the cytoplasmic isoform (SHMT1) in cancerogenesis is currently less defined. In this paper we show that SHMT1 is overexpressed in tissue samples from lung cancer patients and lung cancer cell lines, suggesting that, in this widespread type of tumor, SHMT1 plays a relevant role. We show that SHMT1 knockdown in lung cancer cells leads to cell cycle arrest and, more importantly, to p53-dependent apoptosis. Our data demonstrate that the induction of apoptosis does not depend on serine or glycine starvation, but is because of the increased uracil accumulation during DNA replication.

The alteration of cellular metabolism has been recently recognized as a hallmark of cancer.^1^ Central to the metabolic reprogramming of cancer cells are the complex pathways involving folates, providing the essential precursors to sustain cancer cell growth and affecting cellular antioxidative and methylation capacities, thus supporting tumor homeostasis.^2^ Serine hydroxymethyltransferase (SHMT) is a key protein in this scenario: its main function is to catalyze the folate-dependent serine/glycine interconversion. In the human genome, two *SHMT* genes are found; *SHMT1*, encoding the cytoplasmic isozyme (SHMT1), and *SHMT2*, encoding the mitochondrial one (SHMT2).^3^ *SHMT2* encodes a second transcript that lacks the mitochondrial import sequence, *SHMT2α.*^4^ The SHMT2 isozyme appears to be preferentially involved in the synthesis of mitochondrial thymidine monophosphate (dTMP), however, its chief function is probably to generate one-carbon units from serine, which are exported as formate into the cytosol and support one-carbon metabolism.^5, 6, 7^ Production of glycine inside the mitochondria, required for heme biosynthesis and further production of one-carbon units by the glycine cleavage system, is also a pivotal function of SHMT2.^8,9^ It has been also demonstrated that SHMTs are involved in processes other than enzymatic activity. SHMT1 and, to a lesser extent, SHMT2α participate as scaffold proteins to the synthesis of nuclear dTMP, undergoing nuclear localization in the S-phase during the thymidylate cycle along with thymidylate synthase (TS) and dihydrofolate reductase (DHFR) in order to limit the uracil misincorporation in DNA.^4, 10, 11^

SHMT2 has been shown to be a crucial factor in the serine/glycine metabolism of several cancer cell types, including colon and breast, and thus represents a hot target for selective anticancer drugs.^12^ Lung cancer is the second most common cancer expected to occur in men and women in 2014 and the first leading cause of cancer-associated deaths worldwide.^13^ Recently Zhang *et al.*^14^ demonstrated that SHMT1 is overexpressed in tumor-initiating lung cancer cells, suggesting that the function of this gene could be cancer cell type specific. Here we demonstrate that SHMT1 is upregulated in patient-derived lung cancer tissue samples, there is a crosstalk among SHMT1 and 2 and, unlike other cancer cell types, the downregulation of SHMT1 in lung cancer cell lines through RNAi induces cell cycle arrest and apoptosis, the latter with the involvement of p53. Our results also demonstrate that the antitumor effect induced by SHMT RNAi is not because of serine or glycine starvation, but is mediated by the increased misincorporation of uracil in the genomic DNA possibly because of dTMP loss. Our data open the possibility to target cytoplasmic SHMT1 to treat lung cancer patients.

## Results

### SHMT1 and SHMT2 expression in lung cancer

**Figure 1:**
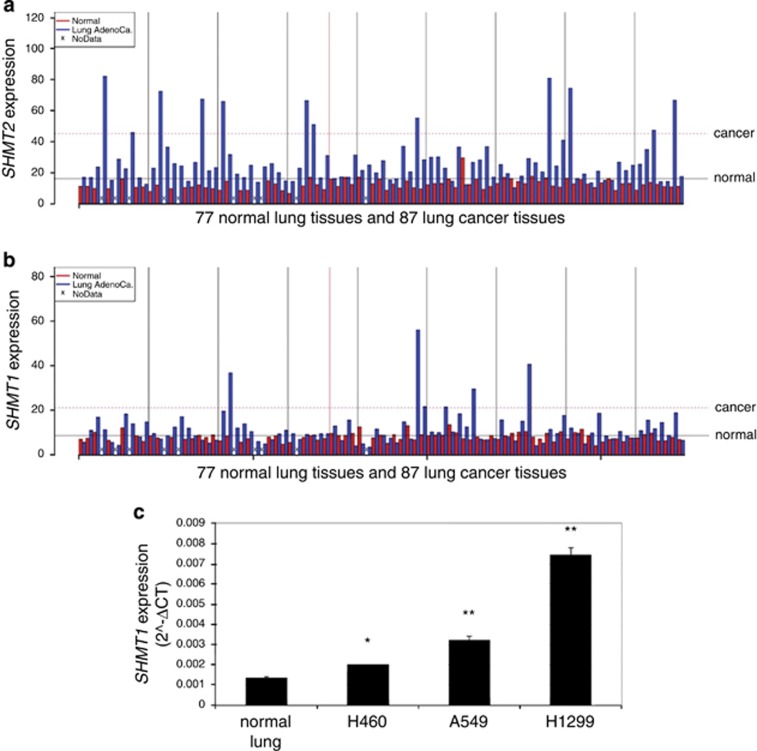
Expression levels of SHMT2 and SHMT1 messenger RNA in samples from lung cancer patients and cell lines. (**a** and **b**) Histogram representing the level of SHMT2 and SHMT1 expression, respectively, on data collected in Gene Expression Omnibus (GEO) database; *t*-test was performed. (**c**) SHMT1 expression level in selected lung cancer cell lines compared with a normal lung sample as measured by quantitative real-time PCR (qRT–PCR), the histogram represent the mean of triplicate samples from three independent experiments with S.D. as error bars, *P≤ 0.05, **P≤0.01

To study the expression of SHMT1 and SHMT2 in lung cancer we used the data collected under the accession number GSE40419 of the NCBI Gene Expression Omnibus (GEO) database to analyze the expression of the two isozymes in the lung cancer tissue collection, compared with the normal counterpart taken from the same patient. SHMT2 is commonly overexpressed in several cancer cell types^12^ and we confirmed these observations in our data set with an average of 2.22-fold increase (*P*=8.7 × 10^−12^; Figure 1a). Surprisingly, as shown in Figure 1b, we also observed an average 1.44-fold increase of *SHMT1* expression in cancer samples (*P*=4.74 × 10^−5^). In parallel, the expression of the *SHMT1* gene was analyzed in three lung cancer cell lines (H460, H1299 and A549) and an upregulation with respect to a normal lung sample was observed (Figure 1c), confirming the trend seen for patients with cancer. The highest levels of *SHMT1* expression were found in A549 and H1299; thus these cells were chosen for a more extensive characterization of the role of SHMT1 in lung cancer.

### RNAi against SHMT1 induces SHMT2*α* upregulation

**Figure 2:**
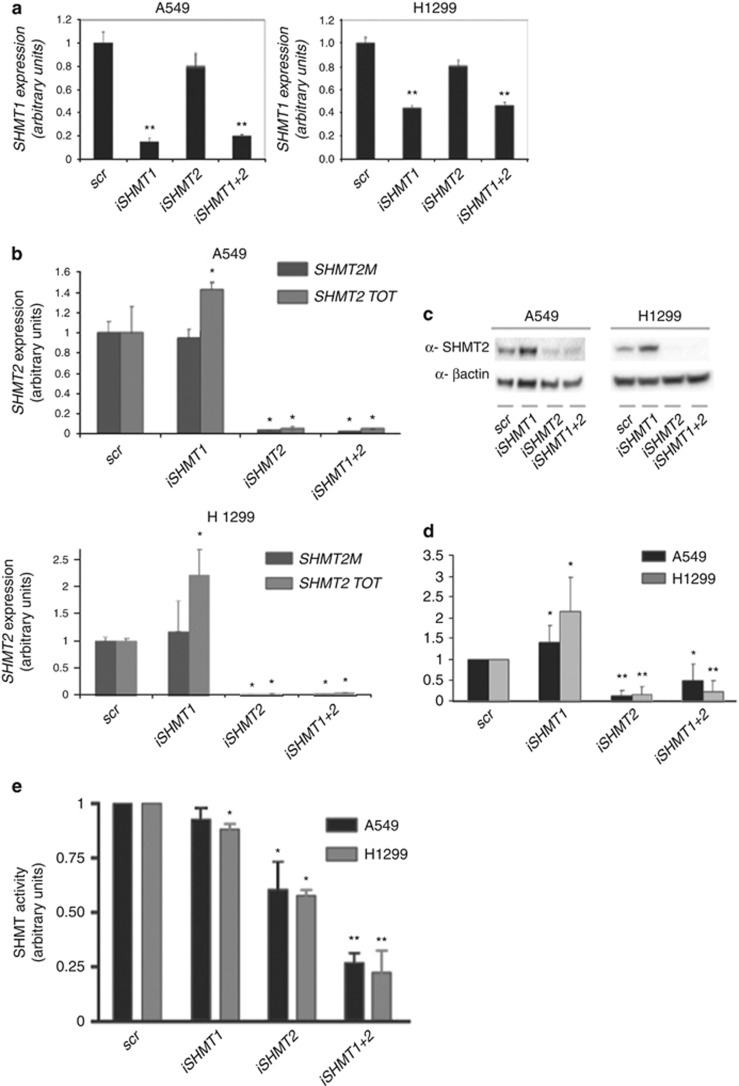
Expression and activity of SHMT isozymes in A549 and H1299 lung cancer cells, measured 48 h after transfection with the indicated RNAi. (**a**) SHMT1 expression level obtained by qRT–PCR and normalized with respect to cells treated with the scrambled sequence (scr). (**b**) Total (SHMT2 TOT) and mitochondrial SHMT2 (SHMT2M) expression level by qRT–PCR. (**c**) Western blot analysis of SHMT2 protein expression; *β*-actin was used as loading control. (**d**) Densitometric analysis performed on SHMT2 Western blot images from three independent experiments. *P≤ 0.05, **P≤0.01. (**e**) Total SHMT enzymatic activity measured by tritium-exchange radioisotopic assay. *P≤ 0.05, **P≤0.01

Considering the previously described importance of *SHMT2* in cancer cells^12, 14^ and our data on *SHMT1* reported in Figure 1, the effect of SHMT(s) depletion in lung cancer cell lines was studied by RNAi (iSHMT). In preliminary tests, the A549 and H1299 cell lines were transfected with a scrambled sequence (scr), or three different iSHMT1 or iSHMT2 sequences. Supplementary Figure 1 demonstrates a similar downregulation effect using the three different RNAi sequences for each gene; for this reason, the three RNAi sequences were used indifferently in the subsequent experiments. Upon transfection, downregulation of about 85% and 50% of SHMT1 mRNA expression was observed in A549 and H1299 cells, respectively (Figure 2a); the same level of downregulation of the iSHMT1-treated cells was observed in cells transfected with iSHMT1+iSHMT2. In order to assess the effect of interference on *SHMT2* mRNA expression, we used a specific set of primers, which enabled us to measure either the total *SHMT2* transcript *(SHMT2 TOT, i.e. SHMT2*+*SHMT2α*) or the mitochondrial *SHMT2* isoform transcript only (Figure 2b). As expected, the iSHMT2 completely knockdowns both isoforms of this gene in both the cell lines. On the other hand, iSHMT1 leaves unaltered the levels of mitochondrial *SHMT2* but, surprisingly, increases total *SHMT2* levels, probably by upregulating the cytoplasmic *SHMT2α* transcript. This observation suggests that a certain level of crosstalk between the two SHMT isoforms (SHMT1 and SHMT2) is operative. Figure 2c and the relative densitometric analysis (Figure 2d) shows the total SHMT2 protein levels (which are actually the sum of SHMT2 and SHMT2*α*) as measured by Western blot in the various knockdowns. The iSHMT2 treatment strongly downregulates the SHMT2 protein in the single and combined (iSHMT1+2) transfections, as expected, on the other hand, iSHMT1 induces the upregulation of the SHMT2 protein levels, confirming that the expression of SHMT1 and SHMT2 isozymes is somewhat interconnected in the lung cancer cell lines that we have analyzed (A549 and H1299).

The SHMT enzymatic activity in total cell extracts was then measured by a radioisotopic assay, with known specificity for SHMT,^15^ which evaluates the exchange of tritium from glycine to water. Figure 2e shows that the iSHMT1 transfection induces only a limited decrease of SHMT activity; these data agree with the observed upregulation of the SHMT2*α* isoform upon treatment with iSHMT1. On the contrary, iSHMT2 induces a larger reduction and, as expected, iSHMT1+iSHMT2 almost completely abolishes SHMT activity.

### iSHMT1 transfection induces cell cycle arrest and apoptosis in lung cancer cell lines

**Figure 3:**
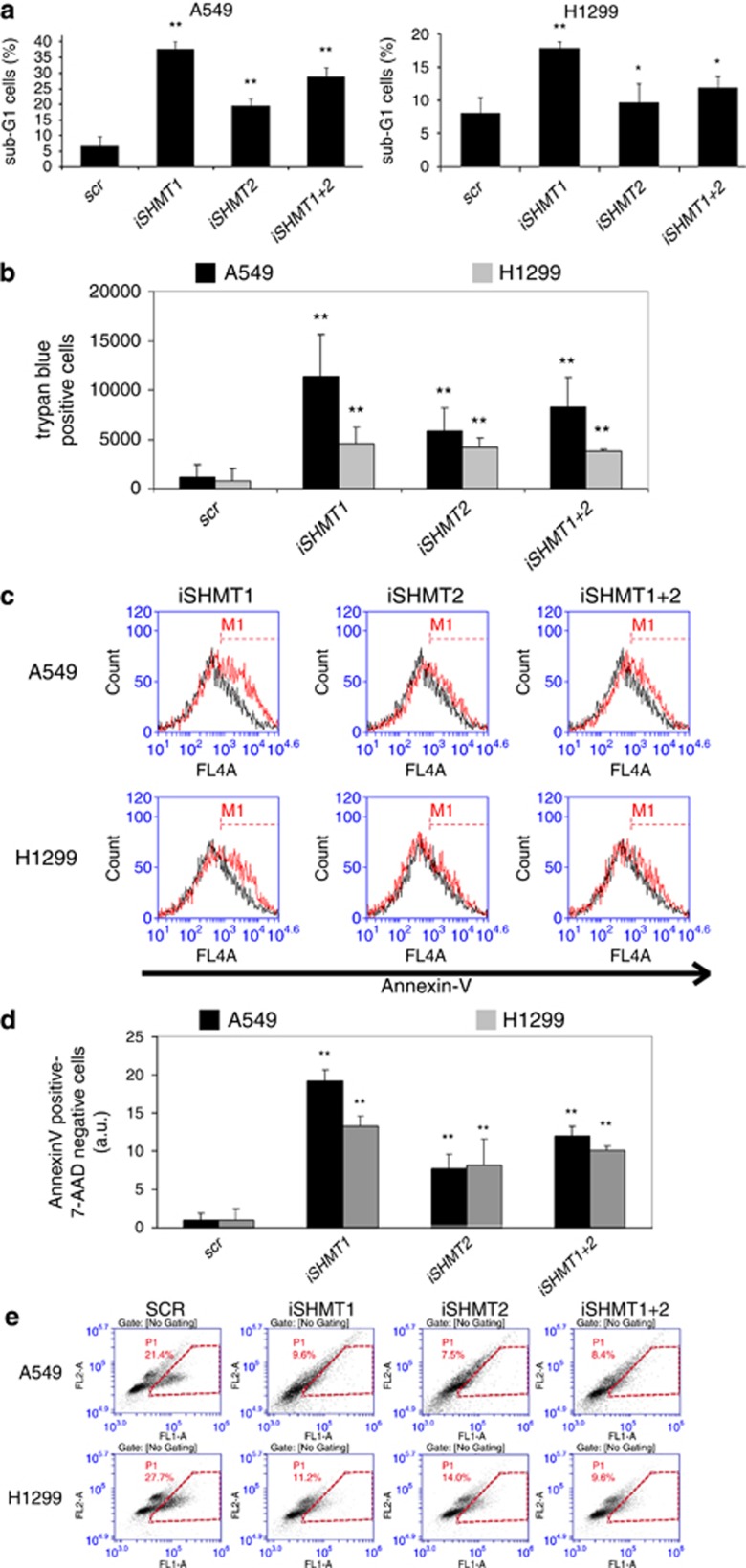
RNAi against SHMT isozymes induce cell apoptosis and cell cycle arrest. A549 and H1299 cells transfected with the indicated RNAi were analyzed for the apoptotic rate after 72 h using PI (**a**) or trypan blue exclusion assay (**b**) and after 48 h using Annexin-V staining (**c** and **d**), the histograms in (**c**) and (**d**) have been produced selecting only 7-AAD negative cells. (**e**) The effects of the transfection of the indicated RNAi on A549 and H1299 cells proliferation were tested by BRDU incorporation assay; cells in S phase fall within the red dashed line. In (**a**, **b** and **d**) *P=0.05, **P=0.01

SHMT2 is upregulated in several cancer cell types and is considered a hot target because its downregulation induces cell cycle arrest.^12^ As, unexpectedly, we have observed that in lung cancer cells SHMT1 is upregulated, we further investigated the effect of its knockdown on the induction of apoptosis and cell cycle arrest. Figure 3a shows that iSHMT1 induces a clear accumulation of a sub-G1 phase population of A549 cells (~33% increase) indicating a strong induction of apoptosis; the effect on H1299 cells is smaller (~10% increase). On the other hand, iSHMT2 induces a significantly lower-apoptotic effect on both the cell lines confirming that, unlike other cancer cell types, SHMT1 has a more important role than SHMT2 in lung cancer cell survival. Surprisingly, the transient knockdown of SHMT1+SHMT2 induces apoptosis to a lower level than the individual SHMT1 knockdown, suggesting that the imbalance between the two isozymes could also be an important determinant in driving cell death. To confirm that those observed in sub-G1 phase are dying cells, we used the trypan blue exclusion assay. Figure 3b confirms that the treatment with the RNAi induce cell death and that the trend is similar to that observed with the propidium iodide (PI) staining in Figure 3a. In order to investigate apoptosis in its early phase and confirming the PI staining data, A549 and H1299 cells were transfected, as described before, and after 48 h were subjected to 7-AAD–AnnexinV-APC staining (Figures 3c and d). We observed an apoptotic induction of 19, 7 and 11% in iSHMT1, iSHMT2 and iSHMT1+2 transfected A549 cells, respectively, as compared with cells transfected with the SCR. The same experiment in the H1299 cell line shows an average increase of the apoptotic rate of 12, 8 and 9% after the iSHMT1, iSHMT2 and iSHMT1+2 transfection, respectively. Interestingly, the samples after iSHMTs transfection show a similar effect on cell cycle arrest in both A549 and H1299 cell lines (Figure 3e), suggesting that knockdown of both SHMT isoforms affects the cell cycle to a similar extent.

### p53 is involved in iSHMT-dependent cell cycle arrest and apoptosis

**Figure 4:**
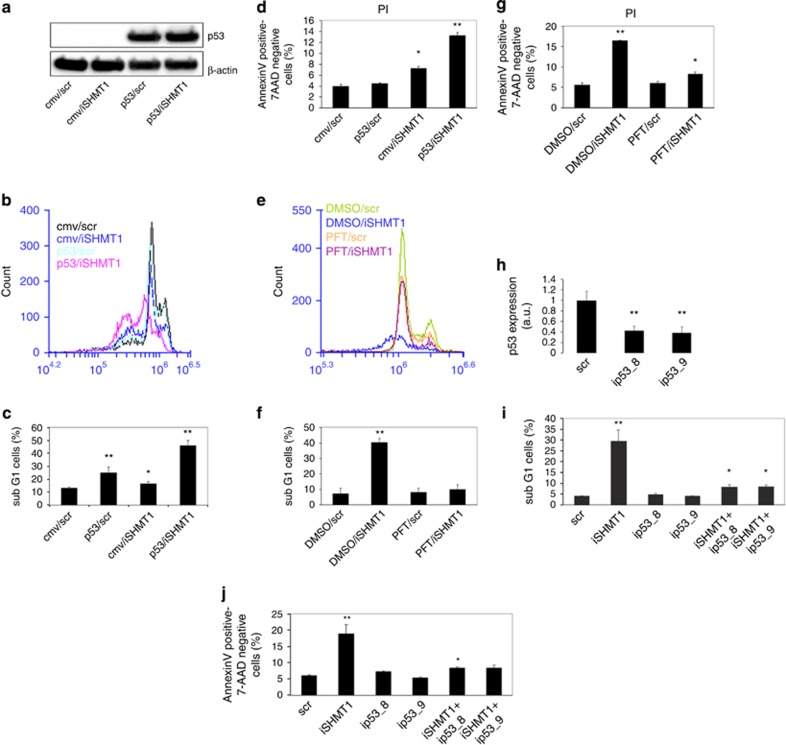
p53 is involved in cell cycle arrest and apoptosis induced by iSHMT1 transfection. (**a**) Western blot analysis using p53 antibody was performed on H1299 cells transfected with the SCR sequence or iSHMT and transfected with the indicated combination of RNAi and plasmids. In panels (**b)** and (**c**), PI staining was employed to estimate the apoptotic rate of H1299 cells transfected with the indicated combination of RNAi and plasmids. In (**d**) the same experiment has been performed using AnnexinV staining to evaluate the apoptotic rate 48 h after the transfection. In (**e**) and (**f**) PI staining was used on A549 cells transfected with the indicated RNAi and treated or not with the p53-inhibitor pifithrin (PFT) 30 *μ*M. In (**g**) the same experiment has been performed using AnnexinV staining to evaluate the apoptotic rate 48 h after the transfection. (**h**) p53 expression was measured by quantitative RT–PCR 72 h after the transfection with RNAi sequences against p53 (ip53_8 and ip53_9; the target sequences are reported in the section Materials and Methods). In (**i**) the PI staining was used on A549 transfected with iSHMT1 in combination or not with two RNAi sequences targeting p53. In (**j**) the same experiment has been performed using AnnexinV staining to evaluate the apoptotic rate 48 h after the transfection. The histograms in (**d**, **g** and **j**) have been produced selecting only 7-AAD negative cells. In (**b**) and (**e**) the result of a representative experiment is shown, whereas the histograms in (**c**, **d**, **f**, **g**, **h**, **i** and **j**) represent the mean of triplicate samples from three independent experiments with S.D. as error bars, *P≤ 0.05, **P≤0.01

It is known that, unlike A549 cells, H1299 cells do not express p53 owing to a deletion of the gene.^16^ As p53 is a well known regulator of apoptotic induction under several conditions,^17^ we hypothesize that the difference in the extent of apoptotic induction between the two cell lines after iSHMT1 knockdown could be owing to the lack of expression of p53 in H1299 cells. To test this hypothesis, we double transfected H1299 cells with iSHMT1 plus a plasmid encoding WTp53 gene or the relative controls (CMV plasmid and scr) and confirmed p53 protein expression (Figure 4a). Figures 4b and c show that 72 h after the double transfection with p53+iSHMT1 the cells undergo a strong apoptotic induction (~35%), whereas transfection with p53 and scr induces only a small increase in the sub-G1 cell population with respect to the control-treated cells (scr+CMV). These data were also validated 48 h after transfection using the AnnexinV staining, observing a similar trend of apoptotic induction (Figure 4d). A well known p53-inhibitor is pifithrin-*α* (PFT), which is able to suppress p53-dependent apoptosis.^18^ We have analyzed the effect of PFT on iSHMT1-treated A549 cells: as shown in Figures 4e–g, addition of PFT significantly decreases the iSHMT1-induced apoptosis. To confirm these data, we used two RNAi sequences against p53 (ip53_8 and ip53_9) in A549 cells obtaining very efficient RNA targeting with both sequences (Figure 4h). We cotransfected iSHMT1 sequence together or not with the two ip53 sequences, then we performed apoptotic analysis with both PI (after 72 h) and AnnexinV (after 48 h) staining. The data in Figures 4i and j confirm that the RNAi mediated p53 knockdown completely revert the iSHMT-induced apoptotic effect. These results clearly indicate that the iSHMT1-induced death signal is mediated by p53.

### iSHMT1-induced apoptosis is not because of serine or glycine starvation

It has been previously proposed that cell cycle arrest induced through SHMT downregulation could be because of the lack of glycine or serine produced by this treatment.^12, 19^

To get more insight into the mechanism whereby interference against SHMT1 is able to induce apoptosis we attempted to rescue cell growth by supplementing these two amino acids in the culture medium. We have transfected A549 and H1299 cell lines with scr or iSHMT1 and after 18 h the transfection medium was replaced by either a minimal medium or minimal medium supplemented with glycine or serine (0.4 mM each). Supplementary Figure 2 shows that there is not a significant difference in apoptosis induction in cells treated with media supplemented with glycine or serine with respect to transfected cells grown in minimal medium. This demonstrates that the apoptotic induction is not owing to iSHMT1-induced glycine or serine starvation.

### iSHMT transfection induces uracil misincorporation in lung cancer cells

**Figure 5:**
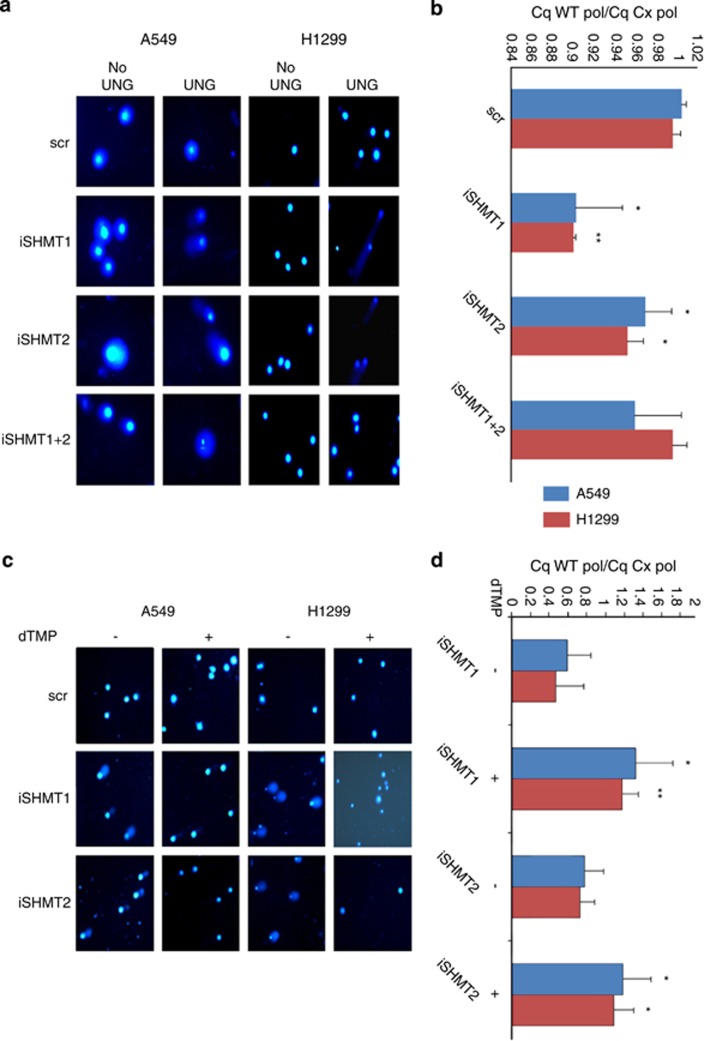

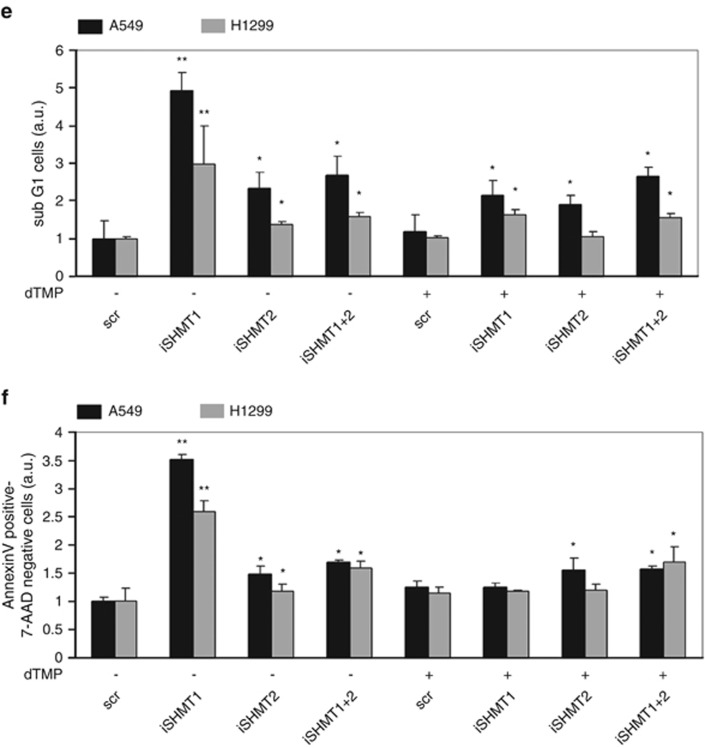
(**a**) Comet assay. The experiment was performed using bacterial uracil DNA glycosilase (UNG) on A549 and H1299 cells transfected with the indicated RNAi. Formation of the comet indicates uracil incorporation in the DNA. The comet assay performed without the digestion with UNG was used as internal control. (**b**) quantitative PCR-based method to quantify uracil incorporation in the DNA of lung cancer cell lines transfected with the indicated RNAi. The ratio among the Cq obtained with uracil-sensitive and the Cq obtained with the uracil-insensitive polymerase is shown. *P≤ 0.05, **P≤0.01. In (**c** and **d**) the comet assay and the quantitative PCR method for uracil incorporation were repeated after the transfection with the indicated sequences and growth in RPMI (-) or RPMI supplemented with dTMP 30 *μ*M (+). In (**e** and **f**) PI and AnnexinV staining respectively were used to estimate the apoptotic rate of A549 and H1299 cells transfected with the indicated RNAi and growth in RPMI (-) or RPMI supplemented with dTMP 30 *μ*M (+). The histograms represent the mean of triplicate samples from three independent experiments normalized with the respect to the cells transfected with the SCR sequence with S.D. as error bars, *P≤ 0.05, **P≤0.01

As previously mentioned, another important function mediated by SHMT is the formation of a TS multienzyme complex needed for the nuclear dTMP production. Mice lacking SHMT1 are vital but have an abnormal accumulation of uracil in DNA.^4^ To test if iSHMT can induce uracil misincorporation in DNA of A549 and H1299 lung cancer cells, we employed two different techniques, namely the comet assay^20^ (performed 72 h after the transfection), which measures the formation of comets in cells treated with bacterial uracil DNA glycosidase, and a qPCR method based on the ability of two commercial polymerases to proceed in the elongation when uracil is erroneously incorporated in the template DNA sequence (performed 48 h after the transfection). Results demonstrate that in iSHMT1 and iSHMT2-transfected cells, Figures 5a and b, respectively, there is misincorporation of uracil in DNA; no significant effect is observed in cells treated with the double interference (iSHMT1+iSHMT2), in accordance with data presented above. In the (more quantitative) qPCR assay, in cells transfected with the scr or with both iSHMT1+iSHMT2, the efficiency of the polymerases is equal and therefore the level of uracil incorporated in the DNA is below the detection limit. On the other hand, in the DNA extracted from cells treated with either iSHMT1 or iSHMT2, the ratio of the Cq obtained with the uracil-sensitive and the uracil-insensitive polymerases is 0.89 and 0.94 for A549 (*P*=0.022 and 0.049), and 0.89 and 0.95 for H1299 (*P*=0.0035 and 0.047), demonstrating that iSHMT1-treated cells incorporate a higher amount of uracil in their genomic DNA. To confirm that the uracil misincorporation in our model is owing to dTMP starvation we tried to rescue this phenomenon repeating the described experiments adding or not dTMP to the culture media of the cells. Figures 5c and d show that the dTMP supplementation suppresses the iSHMT1 and iSHMT2-induced uracil misincorporation. The evidence that uracil misincorporation is occurring in cell lines prompted us to hypothesize that dTMP starvation could represent the mechanism underlying apoptosis induction. To verify this hypothesis, we attempted to rescue cell growth by supplementing dTMP in the culture medium. Figures 5e and f shows that, in the presence of dTMP, the highest reduction in the apoptotic rate is observed in the SHMT1 knockdown whereas a much smaller effect is seen in the iSHMT2-transfected cells, thus clearly showing that induction of apoptosis is owing to iSHMT1-induced dTMP starvation. In agreement with the lack of uracil accumulation (Figure 5a) in the iSHMT1+2 transfected cells, no rescue of cell growth is seen in these samples in presence of dTMP supplementation.

## Discussion

In this study we show that in addition to SHMT2, SHMT1 is overexpressed in samples from lung cancer patients and lung cancer cell lines, suggesting that, at least in this type of tumor, SHMT1 might have a relevant role. We demonstrate that SHMT1 knockdown in lung cancer cells leads to cell cycle arrest and, more importantly, to p53-dependent apoptosis. We further present evidence that SHMT1-dependent apoptosis is because of increased uracil accumulation during DNA replication and probably to a decrease in dTMP synthesis. Detailed experiments to assess the concentration of specific nucleotides/metabolites in situations of SHMT protein imbalance will be crucial to clarify our scenario. Our study uncovers an unforeseen role of SHMT1 in lung cancer cells and, more importantly, opens to the possibility to target this protein for the treatment of lung cancer. Recent studies have highlighted the role of the nonessential amino acids serine and glycine in supporting tumor growth.^21^ Fueling serine into the one-carbon cycle and supporting nucleotide synthesis is thus emerging as a critical pathway in allowing malignant transformation.^22, 23^ SHMTs are at the center of this complex metabolic network controlling not only the levels of amino acids but also of DNA bases (purines and thymidilate) and of the methyl donor S-Adenosylmethionine (SAM).^24^ While the evidence that SHMT2 is overexpressed and has a key role in the metabolism of different cancer types is well established, SHMT1 does not seem to be central in carcinogenesis.^12^ However, SHMT1 is crucial to the *de novo* synthesis of thymidylate, which also involves SHMT2*α*, TS and DHFR. *De novo* thymidylate biosynthesis activity is reduced by 75% in nuclei isolated from *Shmt1*^−/−^ mice.^25^ We have shown that when SHMT1 is knockdown in lung cancer cells, SHMT2α is selectively upregulated. This upregulation is consistent with the attempt of the cells to counteract the loss of SHMT1 and supplant its function as previously suggested.^25^ Previously published data demonstrated that iSHMT2 transfection induces cell cycle arrest and apoptosis in melanoma, breast, colon and ovarian cancer but on the contrary the effects of iSHMT1 are poorly studied.^12^ Our data demonstrate that, in lung cancer cells, iSHMT2 transfection induces cell cycle arrest but has a marginal apoptotic effect, whereas, surprisingly, iSHMT1 transfection induces cell cycle arrest (to the same level of SHMT2), but has also a clear apoptotic effect in both A549 and H1299 cell lines. The extent of apoptosis observed in the two cell lines is different, with the more dramatic effect seen in the A549 cell line (see below). The double transfection iSHMT1+iSHMT2 induces a complete arrest of the cycle but, surprisingly, induces an apoptotic rate lower than iSHMT1, suggesting that the imbalance between the two isozymes may also play a role in the activation of the apoptotic pathway, as a reduced but balanced level of both proteins has a more limited effect. These findings underline the need of a careful evaluation of the role of the SHMT isoforms in different cancer cell types in order to design a successful strategy targeting these proteins. To better understand the complex mechanism that differently regulates iSHMTs-induced apoptosis we also studied p53, known to have a pivotal role in the control of cancer cell proliferation and survival.^17^ The H1299 cell line does not express p53, because of a deletion of this gene, but undergoes cell cycle arrest after iSHMT1, 2 and 1+2 transfection, thereby demonstrating that, in this model, cell cycle arrest is largely p53-independent. On the other hand, the strong apoptotic effect seen in our cell lines is clearly mediated by p53, as demonstrated by complementation and inhibition/knockdown experiments in both cell lines. It has been shown that the block of cell proliferation and apoptosis induced by SHMT2 downregulation can be at least partially reverted by supplementing the growth medium with glycine demonstrating that the major role of this protein is in the serine/glycine conversion.^12^ Our experiments show that supplementation with serine or glycine fails to block the SHMT1-dependent induction of apoptosis, further supporting the idea that SHMT1 has a different and more important role in lung cancer cells other than the production of amino acids. The increased misincorporation of uracil observed in our experiments suggests that the increase in SHMT2*α* can only partially compensate for SHMT1 in the formation of the human nuclear thymidilate synthase complex, as previously suggested in mouse.^25^ Uracil misincorporation is a well known mechanism of cytotoxicity induced, for example, by fluoropyrimidine chemotherapeutic agents including 5-fluorouracil (5-FU).^26^ Our data shows that the supply of exogenous dTMP blocks uracil misincorporation and restores cell viability, thus providing a clear-cut demonstration that the most important role of SHMT1 in lung cancer cells involves its role as scaffold protein in the TS complex during DNA replication. Although considered a hot target for the development of anticancer drugs,^24^ the compartmentalization of SHMT2 in the mitochondrion makes this protein hard to target with specific inhibitors. Our data demonstrate that not only SHMT1 targeting may result in more effective outcomes, but more importantly open to the possibility to work on this more easily targetable protein for the treatment of lung cancer patients.

## Materials and Methods

### Cell lines

A549, H1299 and H460 cancer cells were purchased from ATCC (Manassas, VA, USA) and were grown in RPMI medium supplemented with 2 mM L-glutamine, 100 IU/ml penicillin/streptomycin, and 10% fetal calf serum (FCS; Sigma-Aldrich, St Louis, MO, USA). The rescue experiments were performed in A549 and H1299 cancer cell grown in Miminum Essential Medium Eagle (Sigma-Aldrich) supplemented with 1X MEM Vitamin Solution, 2 mM L-glutamine, 25 mM Glucose, 10% Fetal Bovine Serum dialyzed, 100 IU/ml penicillin/streptomycin and 0.4 mM Serine and/or 0.4 mM Glycine (Sigma-Aldrich).

### Patients' data analysis

Gene expression values for 87 lung adenocarcinomas and 77 adjacent normal tissues have been accessed at http://gene.gmi.ac.kr^27^ and at the NCBI Gene Expression Omnibus (GEO; ACGSE40419^28^). The differences in SHMT1 expression between normal and tumor samples were tested using a paired Student's *t*-test.

### Western blot analysis

Cell lysates were prepared in 8 M urea (Sigma-Aldrich), protein concentration was determined by Biorad Protein Assay (Bio-Rad, Munchen, Germany). Equal amounts of proteins (40 *μ*g) were subjected to SDS–polyacrylamide gel electrophoresis and were transferred onto a nitrocellulose membrane saturated with 5% bovine serum albumin (BSA) in Tris-buffered saline with 0.1% Tween 20. Membranes were incubated with primary antibody for 18 h at 4°C and subsequently with horseradish peroxidase-conjugated secondary antibody for 1 h at room temperature. Membranes were washed with Tris-buffered saline in 0.1% Tween 20 and developed using the chemiluminescence system Chemidoc MP Imaging System (Bio-Rad). Antibody anti-p21, -p53, -SHMT2, -*β*-actin and the secondary antibodies anti-goat and -mouse were from Santa Cruz Biotechnology (Santa Cruz, CA, USA).

### Cells transfection

1 × 10^5^cells/ml were seeded and simultaneously transfected for 18 h with qiagen AllStars RNAi Controls (scrambled sequnces; Qiagen, Hilden, Germany), shmt1 (1_6 CTGCTGTAAATCAGAAGTGTA, 1_7 CTGACGGAGCTGGGCTACAAA, 1_8 CTCCCGTAATCAGGAAGCCAA) and/or shmt2 (2_6 CAGGCGCAGCAAATTCAATTT, 2_7 CCGGGAGATCCCTTACACATT, 2_10 CCCAGCCAACCTGGCCGTCTA) or p53 (p53_8 TTGCAGTTAAGGGTTAGTTTA, P53_9 AAGGAAATTTGCGTGTGGAGT) (GeneSolution siRNA (qiagen)) and/or WTp53 or CMV control plasmid previously described^29^ using Lipofectamine 2000 (Invitrogen, Carlsbad, CA, USA) following the manufacturer instructions.

### RNA extraction and real-time qRT–PCR analyses

Total RNA was extracted using TRIzol reagent (Invitrogen) following the manufacturers instruction and 1 *μ*g was used for the reverse transcription reaction by using a SuperScript First-Strand Synthesis System Kit (Invitrogen). One microliter of the 1 : 50 dilution of the complementary DNA was used for qRT–PCR analysis performed in triplicate for each sample using KAPA Sybr Fast universal qPCR Kit following the manufacturer instructions. Reactions were performed by Stratagene MX3000P (Stratagene, La Jolla, CA, USA). Primer sequences were as follows: *SHMT1*F AGGAAAGGAGTGAAAAGTGTGGAT, *SHMT1*R GACACCAGTGTCGCTCTGGATCTG; *SHMT2*L ATGCTGTACTTCTCTTTGTTTTGG, *SHMT2α*L AGTGATCCTGAGATGTGGGGAGTT, *SHMT2*R AGGATAACCCTCCGAGTACTTGTT; *p53*F TAACAGTTCCTGCATGGGCGGC, *p53*R AGGACAGGCACAAACACGCACC; *β-ACTIN* F AGGATGGCAAGGGACTTCCTG, *β-ACTIN* R AATGTGGCCGAGGACTTTGAT. Human lung total RNA used as control was from Ambion (Austin, TX, USA).

### SHMT activity assay

SHMT activity in cell extracts was measured using a very sensitive and specific radioassay based on the capability of the enzyme to catalyse the exchange of the pro-2S proton of glycine with solvent.^15^ H1299 and H549 lines, were detached using trypsin, resuspended in PBS supplemented with FCS 5% and the cells washed again twice with cold PBS. Cell extracts were prepared from about 1 × 10^5^ cells. Cells were disrupted as previously described.^30^ SHMT activity was determined incubating 0.03 mM [2-^3^H]glycine (2 × 10^9^ dpm *μ*mol^−1^) and 0.5 mM H_4_PteGlu with increasing amounts (2–200 *μ*L) of cell extracts diluted in 70 *μ*L of 20 mM potassium phosphate buffer pH 7.2. Control reactions were performed to correct for background exchange by the addition of 0.5 mM 5-CHO-H_4_PteGlu. Reactions were started by the addition of folate ligand and incubated at 30°C for 2 h. After incubation, reactions were stopped by the addition of 3% (w/v) trichloroacetic acid and treated as previously detailed^15^ so as to remove radiolabeled glycine and measure radioactivity in the solvent. All measurements, performed in duplicate, were normalized on the basis of protein concentration.

### Apoptosis assays

For PI staining, cells were detached with trypsin, washed with cold PBS plus 5% FCS, and then fixed in 70% ethanol for 24 h. After washing with PBS, cells were incubated with 1 *μ*g/ml PI for 3 h at 25 °C before cytometric analysis by BD Accuri C6 Flow Cytometer (Becton Dickinson, Franklin Lakes, NJ, USA). Cells were considered apoptotic when their DNAcontent was <2N (sub-G1 cell population). Results were analyzed with BD Accuri C6 software and were presented as a percentage of specific apoptosis determined using the following formula: ((% apoptotic cells in experimental sample–%apoptotic cells in control sample)/(100–% apoptotic cells in control sample) × 100). For AnnexinV staining: cells were detached with trypsin, washed with PBS–5% FCS and then placed in binding buffer (Pharmingen, San Diego, CA, USA) to which 7-amino-actinomycin D (7-AAD) and AnnexinV-APC (Pharmingen) were added prior to cytometric analysis. Cells were considered apoptotic when Annexin-V-APC positive and 7-AAD negative.

### Trypan blue exclusion assay

The cells were harvested and washed in cold PBS two times, following the addition of a 0.4% (wt/vol) trypan blue solution (Sigma-Aldrich), cells were counted using a Burker chamber (Hirschmann, Germany) under an Axioskop 2 plus microscope (Carl Zeiss Microscopy, Switzerland). Cells staining with trypan blue dye were counted as nonviable.

### Proliferation assay

The incorporation of 5-bromo-2-deoxyuridine (BrdU) into DNA of proliferating cells was measured by a FITC–BrdU flow kit (Pharmingen) according to the manufacturer's protocol. Cells were fixed, permeabilized and total DNA was stained with 7-AAD, followed by cytometric analysis.

### Single cell gel electrophoresis (comet assay)

Single cell gel electrophoresis was performed as previously described.^20^ In brief Cells were washed in cold PBS, detached with trypsin and then washed in cold PBS to a final concentration of 1x10^6^ cells/ml. Cells suspension (10^4^ cells/slide) was embedded in 100 ul of 1% low-melting agarose (Sigma-Aldrich) in PBS and spread onto microscopy slides previously coated with 1% Agarose (Bio-Rad). Cells were lysed in the lysis solution (2.5 M NaCl, 100 mM Na_2_EDTA, 10 mM Tris base, 8 g/l NaOH to pH 10; 1% Triton X-100, 10% dimethyl sulfoxide added prior use) at 4 °C for 1 h or 2 h, depending on the cell line. Each slide was washed three times for 5 min in uracil DNA glycosylase (UNG) buffer (60 mM Tris-HCl, 1 mM Na2EDTA, 0.1 mg/ml Bovine Serum Albumin; pH 8). Therefore, 100 *μ*l of either UNG buffer with DNA glycosylase enzyme (0.1 unit/gel, Roche, Basel, Switzerland) or UNG buffer alone were added and the slides were covered with a glass coverslip. The slides were incubated in a moist atmosphere at 37 °C for 1 h. After enzyme treatment, the slides were placed for 40 min in a tank with cold running buffer (300 mM NaOH, 1 mM Na_2_EDTA, HCl to pH 12.5) before electrophoresis for 20 min at 25 V and 250 mA. The slides was equilibrated with 0.4 M Tris pH 7.5 and dried with cold methanol. DNA was stained with Hoechst 33342 (0.5 mg/ml; Molecular Probes, Leiden, The Netherlands) and pictures were taken using AxioCam Hrc on an Axioskop 2 plus microscope (Carl Zeiss Microscopy) with a 10X magnification.

### DNA extraction and qPCR-based uracil incorporation assay

Total genomic DNA was extracted from the lung cancer transfected cells using DNeasy Blood and Tissue kit (Qiagen, Hilden, Germany). The purified DNA was used in an assay for the quantitative determination of uracil incorporation following a protocol previously described.^31^ DNA was digested by *Bam*H1 restriction enzyme (New England Biolabs, Ipswich, MA, USA) and separated on 1% agarose gel. To isolate the DNA fragments for which the detection primers were designed, the region corresponding to a molecular weight of 1.5 kb was purified with Qiagen Gel Extraction Kit, Qiagen). Reaction mixture was in a final 10 *μ*l volume and contained 0.05 units of PfuTurbo Hotstart (Stratagene; Pfu WT-pol) or PfuTurbo C_x_ Hotstart (Stratagene; Pfu V93Q-pol) the uracil-insensitive polymerase (DNA polymerase; Stratagene), 0.175 *μ*M of each primers, 200 *μ*M of each dNTP (Fermentas, Vilnius, Lithuania), 0.5 *μ*l EvaGreen 20 × (Biotium, San Francisco, CA, USA), 30 nM Passive Reference Dye (Stratagene) and 1 *μ*l of DNA. The primers used were as follows F GTTAGTTCCCTGAGCAGATCCTT, R TAAATGGGTGCAAAGGCTACTTTA. Reactions were performed in the reaction buffer provided by the manufacturer for the PfuTurbo Hotstart or PfuTurbo C_x_ Hotstart DNA polymerases. Real-time PCR reactions were performed in Stratagene MX3000P (Stratagene). If the ratio between the Cq obtained with the Pfu WT-pol and the Cq obtained with the PfuTurbo C_x_ Hotstart was significantly <1 the level of uracil incorporation was considered detectable.

### Statistical analysis

Statistical differences were determined either by Student's *t-*test for paired samples or by one-way analysis of variance followed by Student's *t*-test with the Bonferroni correction. *P*≤0.05 was considered significant.

## Abbreviations

SHMT: serine hydroxymethyltransferase
TS: thymidylate synthase
dTMP: Thymidine monophosphate
Wtp53: plasmid encoding for wild type p53
CMV: empty plasmid with cytomegalovirus promoter
DMEM: Dulbecco's modified Eagle's medium
FCS: fetal calf serum
GEO: Gene Expression Omnibus
DHFR: dihydrofolate reductase
scr: scrambled sequence
RNAi: RNA interference
iSHMT: RNA interference against SHMT
ip53: RNA interference against p53
qPCR: quantitative Polymerase Chain Reaction
Cq: quantification cycle
CT: cycle threshold
SAM: S-Adenosylmethionine
5-FU: 5-fluorouracil
SHMT2M: mitochondrial SHMT2
SHMT2 TOT: total SHMT2
DMSO: dimethyl sulfoxide
Ung: uracil n glycosylase
PI: propidium iodide
