# Supplementary figures and images for: SHMT1 knockdown induces apoptosis in lung cancer cells by causing uracil misincorporation
Source: Cell Death Dis. 2014 Nov 20;5(11):e1525–. doi: 10.1038/cddis.2014.482 (PMC4260740; doi:10.1038/cddis.2014.482)

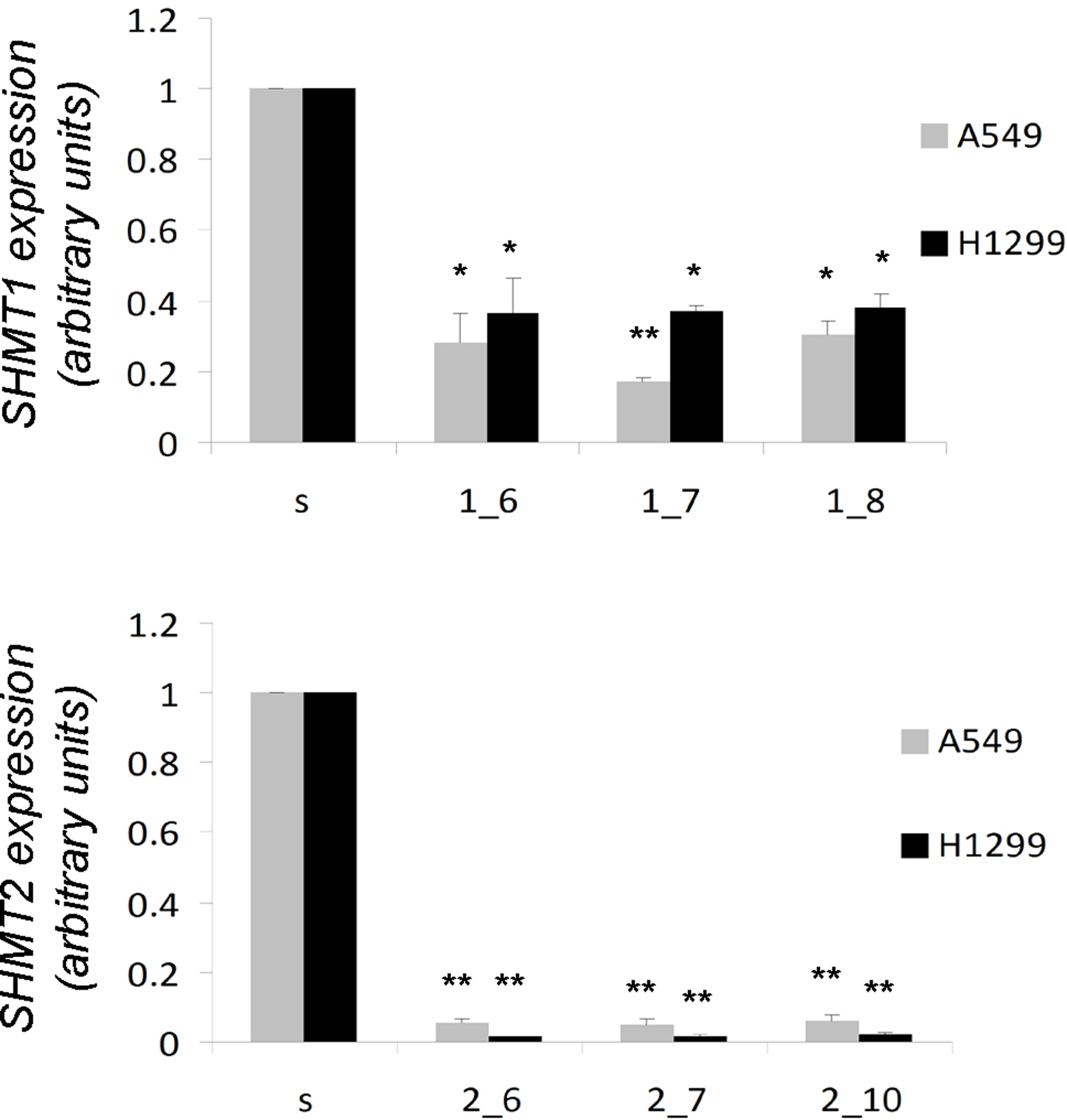

Supplement: Supplementary Figure 1 [file cddis2014482x1.tif]

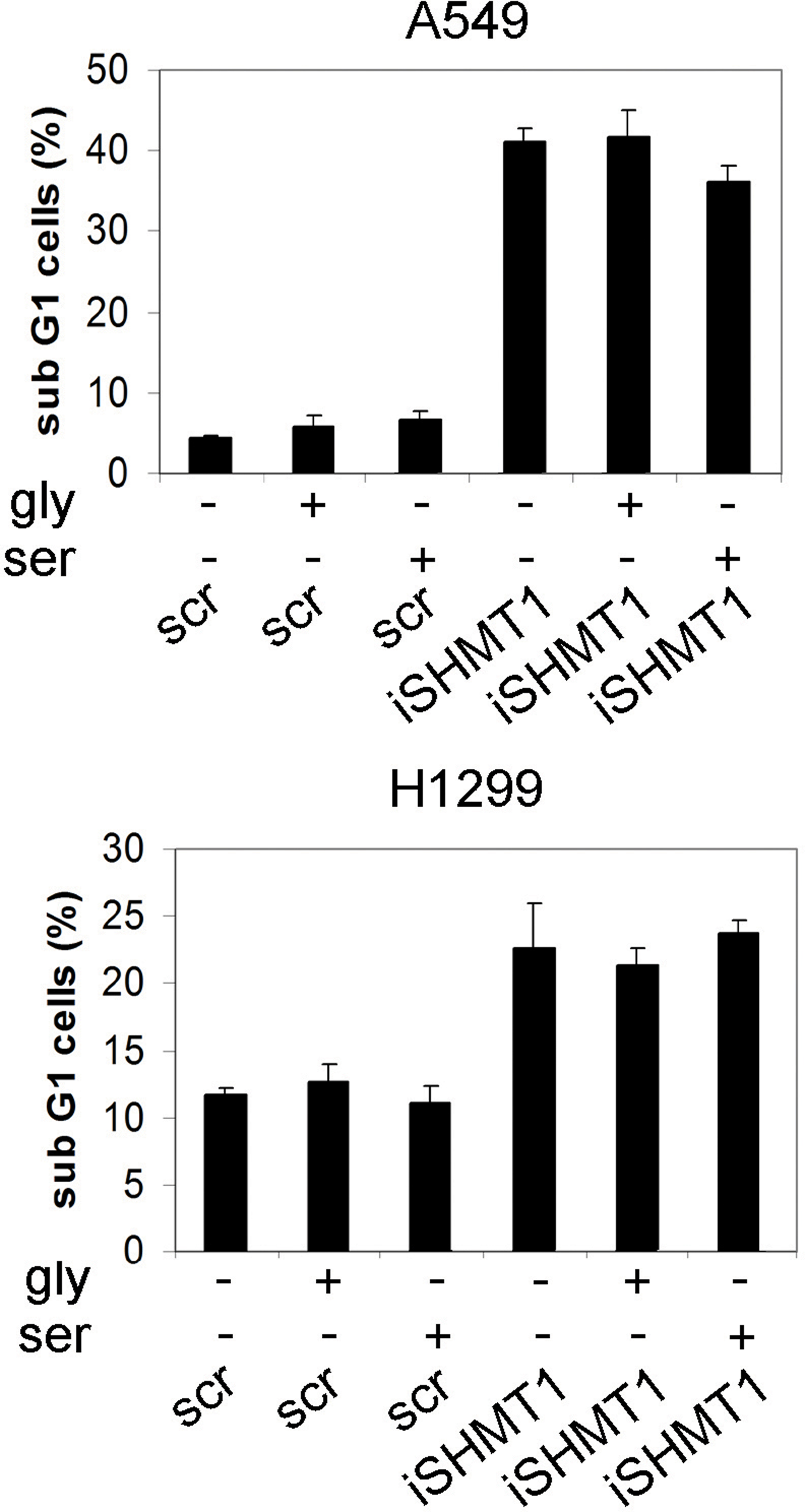

Supplement: Supplementary Figure 2 [file cddis2014482x2.tif]
