# Supplementary material for: SHMT1 knockdown induces apoptosis in lung cancer cells by causing uracil misincorporation
Source: Cell Death Dis. 2014 Nov 20;5(11):e1525–. doi: 10.1038/cddis.2014.482 (PMC4260740; doi:10.1038/cddis.2014.482)
Supplement: Supplementary Figure Legends [file cddis2014482x3.doc]

Supplemental Figure 1

SHMT1 expression level in A549 and H1299 cells transfected with the iSHMT1 (1_6, 1_7 and 1_8) or iSHMT2 (2_6, 2_7 and 2_10) sequences (reported in the materials and methods section) obtained by qRT-PCR and normalized with respect to cells treated with the scr sequence.

Supplemental Figure 2

PI staining was used to estimate the apoptotic rate of the A549 and H1299 cells transfected with the indicated RNAi. Cell growth was carried out using either minimal medium or minimal medium (-) or minimal medium supplemented with serine or glycine (+) as indicated. The histograms represent the mean of triplicate samples from three independent with SD as error bars.
